# Effect of Postoperative Prolonged sedation with Dexmedetomidine after successful reperfusion with Endovascular Thrombectomy on long-term prognosis in patients with acute ischemic stroke (PPDET): study protocol for a randomized controlled trial

**DOI:** 10.1186/s13063-024-08015-x

**Published:** 2024-03-04

**Authors:** Li-na Yang, Yi Sun, Yu-zhu Wang, Jing Wang, Yi-sha Qi, Shan-shan Mu, Yun-peng Liu, Zi-qing Zhang, Zi-mo Chen, Xiao-jie Wang, Wu-xiang Xie, Chang-wei Wei, Yang Wang, An-shi Wu

**Affiliations:** 1 Department of Anesthesiology, Beijing Chao-Yang Hospital, Capital Medical University, Beijing, 100020 People’s Republic of China; 2 Department of Neurosurgery, Beijing Chao-Yang Hospital, Capital Medical University, Beijing, 100020 People’s Republic of China; 3 Department of Neurology, Beijing Tian-tan Hospital, Capital Medical University, Beijing, 100050 People’s Republic of China; 4 Peking University Clinical Research Institute, Peking University Health Science Center, Beijing, 101125 People’s Republic of China

**Keywords:** Dexmedetomidine, Postoperative prolonged sedation, Acute ischemic stroke, Endovascular thrombectomy

## Abstract

**Background:**

Endovascular thrombectomy (EVT) is a standard treatment for acute ischemic stroke (AIS) with large vessel occlusion. Hypertension and increased blood pressure variability within the first 24 h after successful reperfusion are related to a higher risk of symptomatic intracerebral hemorrhage and higher mortality. AIS patients might suffer from ischemia-reperfusion injury following reperfusion, especially within 24 h. Dexmedetomidine (DEX), a sedative commonly used in EVT, can stabilize hemodynamics by inhibiting the sympathetic nervous system and alleviate ischemia-reperfusion injury through anti-inflammatory and antioxidative properties. Postoperative prolonged sedation for 24 h with DEX might be a potential pharmacological approach to improve long-term prognosis after EVT.

**Methods:**

This single-center, open-label, prospective, randomized controlled trial will include 368 patients. The ethics committee has approved the protocol. After successful reperfusion (modified thrombolysis in cerebral infarction scores 2b–3, indicating reperfusion of at least 50% of the affected vascular territory), participants are randomly assigned to the intervention or control group. In the intervention group, participants will receive 0.1~1.0 μg/kg/h DEX for 24 h. In the control group, participants will receive an equal dose of saline for 24 h. The primary outcome is the functional outcome at 90 days, measured with the categorical scale of the modified Rankin Scale, ranging from 0 (no symptoms) to 6 (death). The secondary outcome includes (1) the changes in stroke severity between admission and 24 h and 7 days after EVT, measured by the National Institute of Health Stroke Scale (ranging from 0 to 42, with higher scores indicating greater severity); (2) the changes in ischemic penumbra volume/infarct volume between admission and 7 days after EVT, measured by neuroimaging scan; (3) the length of ICU/hospital stay; and (4) adverse events and the all-cause mortality rate at 90 days.

**Discussion:**

This randomized clinical trial is expected to verify the hypothesis that postoperative prolonged sedation with DEX after successful reperfusion may promote the long-term prognosis of patients with AIS and may reduce the related socio-economic burden.

**Trial registration:**

ClinicalTrials.gov NCT04916197. Prospectively registered on 7 June 2021.

**Supplementary Information:**

The online version contains supplementary material available at 10.1186/s13063-024-08015-x.

## Administrative information

Note: the numbers in curly brackets in this protocol refer to SPIRIT checklist item numbers. The order of the items has been modified to group similar items (see http://www.equator-network.org/reporting-guidelines/spirit-2013-statement-defining-standard-protocol-items-for-clinical-trials/).

**Table:** 

Title {1}	Effect of Postoperative Prolonged sedation with Dexmedetomidine after successful reperfusion with Endovascular Thrombectomy on long-term prognosis in patients with acute ischemic stroke (PPDET): study protocol for a randomized controlled trial
Trial registration {2a and 2b}.	NCT04916197 [ClinicalTrials.gov] [prospectively registered, 7-06-2021] https://clinicaltrials.gov/ct2/show/NCT04916197
Protocol version {3}	Version 1.0 of 2020.4.10
Funding {4}	Capital’s Funds for Health Improvement and Research (grant No. 2022-2Z-2039) (to Changwei Wei).
Author details {5a}	Li-na Yang: Department of Anesthesiology, Beijing Chao-Yang Hospital, Capital Medical University, Beijing, China Yi Sun: Department of Anesthesiology, Beijing Chao-Yang Hospital, Capital Medical University, Beijing, China Yu-zhu Wang: Department of Anesthesiology, Beijing Chao-Yang Hospital, Capital Medical University, Beijing, China Jing Wang: Department of Anesthesiology, Beijing Chao-Yang Hospital, Capital Medical University, Beijing, China Yi-sha Qi: Department of Anesthesiology, Beijing Chao-Yang Hospital, Capital Medical University, Beijing, China Shan-shan Mu: Department of Anesthesiology, Beijing Chao-Yang Hospital, Capital Medical University, Beijing, China Yun-peng Liu: Department of Neurosurgery, Beijing Chao-Yang Hospital, Capital Medical University, Beijing, China Zi-qing Zhang: Department of Neurosurgery, Beijing Chao-Yang Hospital, Capital Medical University, Beijing, China Zi-mo Chen: Department of Neurology, Beijing Tian-tan Hospital, Capital Medical University, Beijing, China Xiao-jie Wang: Department of Anesthesiology, Beijing Chao-Yang Hospital, Capital Medical University, Beijing, China Wu-xiang Xie: Peking University Clinical Research Institute, Peking University Health Science Center, Beijing, China Chang-wei Wei: Department of Anesthesiology, Beijing Chao-Yang Hospital, Capital Medical University, Beijing, China Yang Wang: Department of Neurosurgery, Beijing Chao-Yang Hospital, Capital Medical University, Beijing, China An-shi Wu: Department of Anesthesiology, Beijing Chao-Yang Hospital, Capital Medical University, Beijing, China
Name and contact information for the trial sponsor {5b}	Investigator initiated clinical trial. Changwei Wei (Principal investigator) Changwei.wei@ccmu.edu.cn
Role of sponsor {5c}	This is an investigator initiated clinical trial, the funders played no role in the design of the study and collection, analysis, and interpretation of data and in writing the manuscript.

## Introduction

### Background and rationale {6a}

Stroke is the second-leading cause of death globally, and 62.4% of incident strokes are acute ischemic stroke (AIS) [1, 2]. It is also the main cause of disability, which could lead to a high risk of poststroke cognitive impairment and dementia [3, 4]. Endovascular thrombectomy (EVT) is the standard treatment for AIS patients with large vessel occlusion [5]. The modified thrombolysis in cerebral infarction (mTICI) is an indicator to evaluate recanalization after EVT, ranging from 0 (no reperfusion) to 3 (complete reperfusion). The scale of 2b–3 indicates reperfusion of at least 50% of the affected vascular territory, which is defined as successfully recanalized [6]. The functional outcome of AIS patients is assessed by the categorical variable of the modified Rankin Scale (mRS), ranging from 0 (no symptoms) to 6 (death), and mRS 0–2 indicates functional independence. Although the successful reperfusion rate is about 70–83%, only 40–55% of patients achieved a functional independence outcome [7, 8]. In addition, patients with large ischemic cores might show progressive edema, malignant infarctions, and secondary hemorrhage after recanalization [9–11]. Besides, up to 38.5% of AIS patients could experience emergency agitation, acute hypertensive response, and increased blood pressure variability (BPV) [12]. Previous studies found that hypertension and increased BPV within the first 24 h after EVT were related to a higher risk of symptomatic intracerebral hemorrhage which requires hemicraniectomy and could lead to a higher mortality rate [13–15].

Dexmedetomidine (DEX) is a highly selective α2-adrenoceptor agonist, which is commonly used as a sedative agent during EVT [16, 17]. DEX has dose-dependent properties of sedation, analgesia, and anxiolytic with minimal respiratory depression [18–20]. In addition, DEX could also prevent postoperative agitation and promote cardiovascular stabilization by inhibiting sympathetic nervous system activity [21, 22]. Previous studies found that AIS patients might suffer from ischemia-reperfusion injury following recanalization, especially within 24 h after EVT [23]. Several animal studies reported that DEX could provide neuroprotective effects against ischemia-reperfusion injury by reducing mitochondrial damage [24], attenuating oxidative stress [25], decreasing inflammatory factors [26], and inhibiting neuronal autophagy [27]. Taken together, DEX exerts beneficial effects in the time course of cerebral ischemia-reperfusion and the postoperative period [28].

Therefore, we hypothesize that postoperative prolonged sedation with DEX might provide consistent neuroprotection to rescue the affected vascular territory from further deterioration and promote the functional outcomes for AIS patients undergoing EVT. There is a need to explore the efficacy and safety of postoperative prolonged sedation with DEX on the functional outcome of AIS patients undergoing EVT.

### Objectives {7}

This study aims to investigate whether postoperative prolonged sedation with DEX could:

1. Promote functional independence (mRS 0–2) at 90 days after successful reperfusion
2. Relief of the stroke severity at 24 h and 7 days (or discharge if earlier) after EVT compared with admission, measured with the National Institute of Health Stroke Scale (NIHSS), which ranges from 0 to 42, with higher scores indicating greater severity
3. Rescue the affected vascular territory from further deterioration by assessing ischemic penumbra volume/infarct volume with neuroimaging
4. Reduce the length of ICU/hospital stay and lower the all-cause mortality rate at 90 days

### Trial design {8}

The study is a single-center, open-label, prospective, randomized controlled trial comparing the long-term prognosis of functional outcomes of AIS patients undergoing EVT. This is the first study as far as we know to focus on the postoperative neuroprotective effects of sedatives. Previous studies found that DEX could prevent postoperative agitation, promote cardiovascular stabilization, and alleviate ischemia-reperfusion injury [21–28]. We hypothesize that sedation with DEX after EVT might provide neuroprotective effects and promote the long-term prognosis of AIS patients, by the properties of DEX.

A total of 368 patients after successful reperfusion with EVT will be enrolled and randomly allocated to receive 24 h of DEX as postoperative prolonged sedation or 0.9% saline solution in a 1:1 ratio. The investigator will examine the functional outcomes, adverse events, and all-cause mortality at 90 days and compare the stroke severity and affected vascular territory at 24 h and 7 days (or discharge if earlier) with admission.

**Fig. 1:**
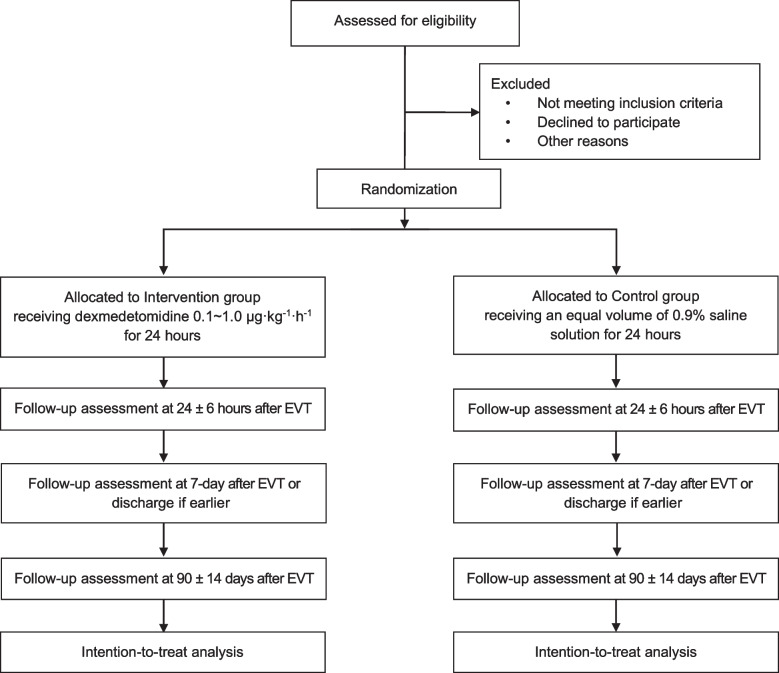
Flowchart of the PPDET trial

The protocol follows the Standard Protocol Items: Recommendations for Interventional Trials (SPIRIT) reporting guidelines for clinical trials [29]. and fully consistent with the Declaration of Helsinki. All items about the WHO Trial Registration Data Set can be found within the protocol. The flowchart of the study is shown in Fig. 1.

### Methods: participants, interventions and outcomes

#### Study setting {9}

The study will be conducted in Beijing Chao-Yang Hospital affiliated with Capital Medical University, which is a large-scale, tertiary hospital in China.

#### Eligibility criteria {10}

Participants must fulfill the criteria shown in Table 1 to be eligible for the trial.

**Table 1:** Inclusion criteria and exclusion criteria of the PPDET trial

**Inclusion criteria**	
(1) 18 years to 80 years	
(2) 2 ≤ NIHSS ≤ 25 [7, 30]	
(3) mRS before stroke less than 3	
(4) AIS (anterior circulation) scheduled for EVT	
(5) mTICI after EVT reaches 2b–3	
(6) Informed consent was signed by patients or legal representatives	
**Exclusion criteria**	
(1) Intracerebral hemorrhage occurred in the responsible vessel area in the past 6 weeks	
(2) Patients who had received stent treatment at the responsible vessel in the past	
(3) Neurological function was restored at or before angiography	
(4) Patients who are allergic to heparin, aspirin, clopidogrel, rapamycin, lactic acid polymer, poly (n-butyl methacrylate), stainless steel, anesthetics, and contrast agents or have contraindications	
(5) Hemoglobin was less than 70 g L^−1^, platelet count was less than 50 × 10^9^ L^−1^, international normalized ratio (INR) greater than 1.5 (irreversible), or uncorrectable bleeding factors	
(6) Blood glucose < 2.7 mmol L^−1^ or > 22.2 mmol L^−1^	
(7) Severe liver or kidney dysfunction, ALT > 3 times the upper limit of normal value or AST > 3 times the upper limit of normal value, creatinine > 1.5 times the upper limit of normal value	
(8) Pregnant or lactating women	
(9) Previous history of mental illness	
(10) Stroke combined with other acute diseases or postoperative stroke of other operations	
(11) Heart rate (HR) less than 50 bpm, a second or third degree of an atrioventricular block (except for pacemaker implantation), and one vasoactive drug cannot maintain the systolic blood pressure (SBP) above 90 mmHg	

#### Who will take informed consent? {26a}

Our Stroke Rapid Response Team will screen potential AIS patients who plan to receive EVT for eligibility criteria to participate in the study in the emergency. The recruiting members from the Stroke Rapid Response Team will complete the informed consent process with any participant meeting the eligibility criteria. The participants or their authorized family members will sign the informed consent and receive a letter of assessment schedule before EVT.

#### Additional consent provisions for collection and use of participant data and biological specimens {26b}

Not applicable since no biological specimens will be collected in this study. The potential for secondary use of data is explained in the initial consent form.

### Interventions

#### Explanation for the choice of comparators {6b}

We designed this study with the expectation of comparing the postoperative prolonged sedation group with the no prolonged sedation group. The two groups receive the same standard of care in ICU. We hope to minimize the potential bias between the two groups by only dissolving DEX to the saline in the intervention group, with both groups considering receiving about the same fluid therapy. Therefore, the control group receives an equal volume of 0.9% saline solution instead of DEX for 24 h.

### Intervention description {11a}

#### Baseline preoperative visits

Baseline assessments are conducted in the emergency. The following baseline characteristics will be collected: age, gender, weight, height, education, at least two telephone numbers (to reduce the dropout rate of telephone interviews), medical history (especially hypertension, diabetes, atrial fibrillation, hyperlipidemia, heart failure), and history of smoking and drinking. NIHSS and mRS (pre-onset and post-onset) are assessed before EVT. Neuroimaging examinations such as computed tomography (CT), CT angiography (CTA), or CT perfusion (CTP) will be collected from electronic medical records. Vital signs including blood pressure (BP), electrocardiography (ECG), blood oxygen saturation, and respiratory rate will be collected from monitors. The eligibility and baseline evaluations will be recorded in a web-based electronic data capture (EDC) system.

#### Intraoperative protocols

Participants in this trial will be transferred from the emergency to the operation room. Standard monitoring including BP, ECG, and oxygen saturation is measured upon arrival at the operation room. Oxygenation is performed through a mask and an intravenous line is established at the forearm. Certified and experienced anesthesiologists conduct anesthesia for all the participants. In our center, local anesthesia with DEX sedation is standard intraoperative anesthesia management for EVT. Participants in both groups receive a loading dose of DEX for 10–15 min, ranging from 0.5 to 1.0 μg kg^−1^, and then maintain 0.5 to 1.0 μg kg^−1^ h^−1^ to the end of EVT. The infusion rate is adjusted according to age, general conditions, complications, BP, and surgical needs. The partial pressure of end-tidal carbon dioxide is maintained between 35 and 45 mmHg. BP is monitored every 5 min. The systolic blood pressure (SBP) is maintained between 140 and 180 mmHg, with the mean arterial pressure (MAP) ≥ 70 mmHg [31]. Vasoactive drugs (e.g., epinephrine, norepinephrine, ephedrine, or isosorbide mononitrate) will be administered to maintain BP properly. Hemodynamic and ventilation abnormalities (e.g., hypertension, hypotension, hypoxemia), vasoactive drug administration, and recanalization time are recorded during EVT. Randomization is performed immediately after the mTICI scale is assessed after EVT. Before transferring to the ICU, neuroimaging exams participants for signs of acute hemorrhagic cerebrovascular events and other EVT-related adverse events.

#### Intervention and control

After arrival at the ICU, participants in the intervention group will receive DEX 0.1–1.0 μg kg^−1^ h^−1^ for 24 h and maintain light to moderate sedation measured by the Ramsay sedation score. The Ramsay sedation score is ranging from 1 (agitated) to 6 (unarousable), and a score of 2–3 is considered a light-moderate level of sedation [18]. Oxygen will be delivered using a nasal catheter at a flow rate of 2–5 L min^−1^. For participants with successful reperfusion, SBP will be maintained between 120 and 180 mmHg, with diastolic blood pressure (DBP) lower than 105 mmHg [32–34].

In the control group, participants will receive an equal volume of 0.9% saline solution instead of DEX for 24 h postoperatively. If the Ramsay sedation score is 1, propofol will be administrated to maintain the Ramsay sedation score at 2–3. The propofol administration (number of applications, duration, and total dose) will be recorded for post hoc analyses.

#### Follow-up visits

During hospitalization, the research team will assess the stroke severity with NIHSS at 24 h and 7 days (or discharge if earlier) after EVT. Neuroimaging includes magnetic resonance imaging-diffusion weighted imaging (MRI-DWI) or CTP examines the ischemic penumbra volume and infarct volume at 7 days (or discharge if earlier). Acute hemorrhagic cerebrovascular events and other adverse events related to EVT or DEX will be collected and recorded through the EDC system.

After discharge, participants or their relatives are followed up by telephone. The survival status, functional outcome, and adverse events will be assessed at 90 days after EVT. The scale of mTICI, mRS, NIHSS, and Ramsay sedation score are presented in Additional file 1.

### Criteria for discontinuing or modifying allocated interventions {11b}

Participants and their authorized family members can withdraw from the study at any time without any negative consequences attached. When participants leave the study, inverse probability weighting and worst-case imputation schemes will be used to process the missing data as part of the sensitivity analyses.

The pre-defined serious adverse event (SAE) related to DEX is based on previous studies and the theory of procedures [35–37]. When the DEX infusion rate is adjusted to the lowest study rate (0.1 μg kg^−1^ h^−1^), SAE is defined if the following situation occurs, including severe respiratory depression (pulse oxygen saturation less than 90%, or a decrease of more than 10% from baseline), severe bradycardia (HR less than 40 bpm or a decrease of more than 30% from baseline), severe hypotension (SPB less than 80 mmHg or a decrease of more than 30% from baseline, or DBP less than 50 mmHg), severe tachycardia (HR more than 120 bpm or an increase of more than 30% from baseline), and severe hypertension (SBP less than 180 mmHg or an increase of more than 30% from baseline, or DBP more than 100 mmHg). Intervention for respiratory depression included the administration of oxygen or endotracheal intubation. Intervention for bradycardia, tachycardia, and hypertension included administration of medication. Intervention for hypotension includes adjustment of medicine and intravenous fluid treatment. The other SAE includes a progression of ischemic stroke, hemorrhagic complications, new ischemic stroke to the different vascular territory, and death.

The study will be prematurely suspended in case of any intolerable and untreatable SAE occurrences related to the DEX or if the independent and experienced neurosurgeon or anesthesiologist advises (safety supervisors). Criteria of termination include two or more intolerable and untreatable SAEs related to DEX. Safety supervisors will screen for SAEs and assess if it is related to the intervention. The final decision on whether suspense or termination of the study rests with the principal investigator (PI).

### Strategies to improve adherence to interventions {11c}

To improve adherence, participants and their authorized family members will be fully explained the intervention, potential risks, and other details by the recruiting members and will be informed of the significance of completing follow-up assessments. They can reach us whenever they have any questions or need medical attention.

### Relevant concomitant care permitted or prohibited during the trial {11d}

All participants will receive standard postoperative management after the EVT procedure. Patients with postoperative pain will be allowed to use analgesic medication.

### Provisions for post-trial care {30}

There will be no provision for patients who participate in the trial, and the post-trial care will follow the standards of care after EVT.

### Outcomes {12}

#### Primary outcome

The primary outcome is the functional outcome at 90 days after EVT. The functional outcome is measured by mRS, which is a 7-grade categorical scale ranging from 0 (no symptoms) to 6 (death). A score of 0–2 indicates functional independence. The mRS will be evaluated by telephone at 90 days after successful reperfusion with EVT. To improve the follow-up rate, we defined 90 days as 90 ± 14 days after EVT to consider situations such as holidays and line occupancy.

#### Secondary outcome

The secondary outcomes include the changes in the stroke severity measured by the continuous variable of NIHSS at 24 h and 7 days compared with admission, the length of ICU stays, the length of hospital stays, and the all-cause mortality rate at 90 days. Neuroimaging outcomes include the changes in ischemic penumbra volume and infarct volume at 7 days compared with admission. The 24 h is defined as 24 ± 6 h after EVT, and 7 days is defined as 7 days or discharge if earlier after EVT.

Safety variables include a progression of ischemic stroke, hemorrhagic complications, new ischemic stroke to the different vascular territory, SAEs related to DEX, and death. Symptomatic intracranial hemorrhage is defined as neurologic deterioration (at least a 4-point increase in NIHSS) with intracranial hemorrhage evidence on the neuroimaging scan. The adverse events/harms are reported by the resident research physician when participants are in the hospital; the survival status and adverse events are collected by follow-up visits through telephone after discharge.

#### Participant timeline {13}

**Fig. 2:**
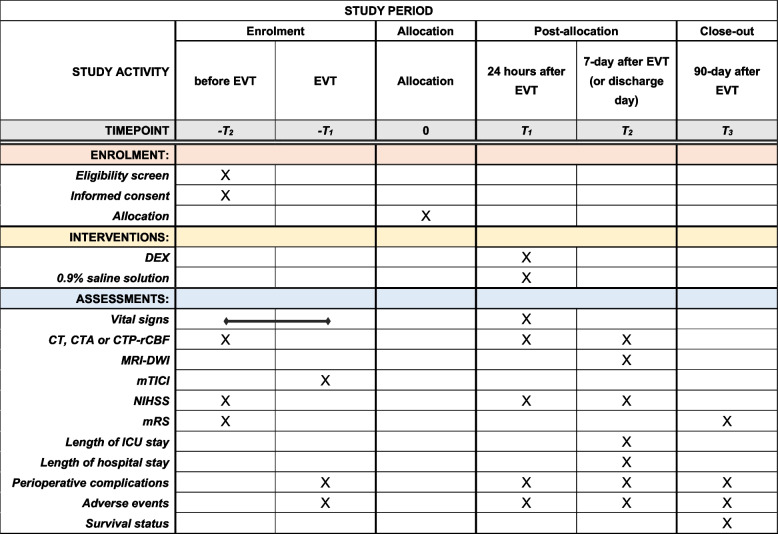
The schedule of enrolment, interventions and assessments of the PPDET trial

The participant timeline is shown in Fig. 2.

#### Sample size {14}

According to previous studies, the functional independence rate at 90 days after EVT for AIS is about 40 to 55% [7, 8]. Given the risk and benefit of infusion with DEX after EVT, at least an increase of 15% in the proportion of mRS 0–2 in the AIS patients with postoperative prolonged sedation is considered clinically meaningful. Thus, we assume 50% of participants in the control group could achieve mRS 0–2 and an increase of 15% in the intervention group. With a power of 80% and 2-side *α* = .05, 167 patients per group are required. Considering a 10% dropout rate and random block length, the total sample size is 368 (184 patients per group). The sample size calculation was conducted on PASS software, version 14.0 (Number Cruncher Statistical Software).

#### Recruitment {15}

Participants are recruited in the emergency of Beijing Chao-Yang Hospital, where annually about 1000 EVT procedures for AIS in the hospital.

### Assignment of interventions: allocation

#### Sequence generation {16a}

Randomization is performed immediately after the mTICI scale is evaluated. Research members use a web-based EDC system to perform randomization. Participants are randomly assigned to the intervention or control group at a 1:1 ratio. A random block length scheme is used to reduce selection bias.

#### Concealment mechanism {16b}

The EDC system stores the results of randomization and personal information with limited access to ensure that the allocation is concealed for mRS evaluators, statisticians, and radiologists.

#### Implementation {16c}

After signing the informed consent forms, the recruiting members from the Stroke Rapid Response Team will use the EDC system to allocate the participants to one of the study arms. The EDC system will generate a random numerical series, with one encoding for the intervention group and zero for the control group.

### Assignment of interventions: blinding

#### Who will be blinded {17a}

Patients and researchers responsible for postoperative evaluation during admission will not be blinded since it is easy to distinguish these two groups according to the postoperative prolonged drowsiness induced by DEX. But investigators responsible for mRS evaluations at 90 days, statisticians, and radiologists are blinded to the allocation.

#### Procedure for unblinding if needed {17b}

This is an open-label trial. Therefore, there is no unblinding procedure.

### Data collection and management

#### Plans for assessment and collection of outcomes {18a}

All team members responsible for assessment are trained and obtained certification from the official training program website (www.nihstrokescale.org) before evaluating NIHSS and mRS. During hospitalization, the NIHSS and adverse events will be assessed 24 h and 7 days after EVT, while at 90 days after EVT, the mRS, survival status, and adverse events will be assessed with other blinded evaluators by telephone interview. The neuroimaging will also be evaluated by blinded and independent radiologists. The hospital/ICU stay will be directly collected from the electronic patient records. Data are entered into the EDC system with an electronic case report form (eCRF).

#### Plans to promote participant retention and complete follow-up {18b}

The research team will make every attempt to follow the participants throughout the study period. If the participant discontinues the study (e.g., withdraws the consent or loses to follow-up), the reason will be recorded as follows. Several strategies are used to enhance participant retention, including keeping participants informed of the upcoming data collection by providing adequate communication when visiting participants or authorized family members, providing a letter about the assessment schedule before data collection to remind them of each assessment, and ensuring participants that they can continually assess for other outcomes if they withdraw for a follow-up visit.

#### Data management {19}

Data are collected with an eCRF on the EDC system. This web-based system can automatically perform range checks based on pre-defined logic rules when data enters. The eCRF can record any changes made to the raw data. In case of information loss, backups will be made regularly every 3 months to a hard drive. Signed informed consent forms will be stored in a locked cabinet, and according to the guidelines for Advanced Therapy Medicinal Products (ATMPs), all research data will be retained for 30 years after the termination of the study.

#### Confidentiality {27}

Data will be converted into numeric codes for anonymous processing and subsequent procedures. The decoding list will only be available to the researcher during the study period and will be held by the PI. No details related to personal information will be reported in public.

#### Plans for collection, laboratory evaluation and storage of biological specimens for genetic or molecular analysis in this trial/future use {33}

Not applicable since no biological specimens were collected in this study.

### Statistical methods

#### Statistical methods for primary and secondary outcomes {20a}

Continuous variables will be presented as either mean (± standard deviation, SD) or median (interquartile range, IQR), depending on the distribution. Group comparisons for continuous variables will be conducted using either analysis of variance (ANOVA) for parametric data or a nonparametric test, considering the distribution of the data. Categorical variables will be expressed as numbers and percentages. Group comparisons for categorical variables will be performed using the *χ* ^2^ test or Fisher exact probability test, depending on sample size and data distribution.

The primary outcome involves comparing the functional independence rate (mRS 0–2) at 90 days after successful reperfusion between groups. We will employ the modified Poisson regression model to analyze the relative risk (RR) and estimate a 95% confidence interval (CI) for the comparison between groups. The choice of the modified Poisson regression model is based on its suitability for estimating relative risks for binary outcomes in cohort studies [38].

For secondary outcomes, the *χ* ^2^ test or Fisher exact probability test will be used to compare group differences. Secondary outcomes may include additional clinical or functional measures not addressed in the primary analysis.

Sensitivity analyses will be performed to assess the robustness of the findings and explore potential variations in the results under different assumptions or analytical approaches.

Statistical significance will be set at *P* < 0.05, with all tests being two-tailed. The statistical analyses will be conducted using SPSS software, version 22.0 (SPSS, Inc).

#### Interim analyses {21b}

No interim analyses nor efficacy and safety interim analyses are planned, but we will monitor and collect the adverse events case by case.

#### Methods for additional analyses (e.g., subgroup analyses) {20b}

There are no subgroup analyses planned.

#### Methods in analysis to handle protocol non-adherence and any statistical methods to handle missing data {20c}

We will perform analyses according to the intention-to-treat principle. The participants are included in the final analysis after randomization. Per-protocol analysis can be used as a sensitivity analysis to supplement the results of the main analysis. Missing data will be adjusted using inverse probability weighting and worst-case imputation schemes.

#### Plans to give access to the full protocol, participant-level data, and statistical code {31c}

The datasets are available from the correspondence author (An-shi Wu, wuanshi88@163.com) upon reasonable request.

### Oversight and monitoring

#### Composition of the coordinating center and trial steering committee {5d}

The study is a single-center, open-label, prospective, randomized controlled trial performed and coordinated in the Beijing Chao-Yang Hospital, with the collaboration of the Department of Anesthesiology and Neurosurgery. Day-to-day support for the trial is organized as follows:

- PI: supervises this trial, safeguards the randomization decoding files and the backup hard drive, reports adverse events to the steering committee and ethics committee, determines whether suspense or termination of the case/study, and takes responsibility for the participants
- Stroke Rapid Response Team: screens potential participants, takes informed consent, performs EVT, reports adverse events, and conducts randomization online
- Resident research physician: processes intervention/control treatment according to protocol, reports adverse events, and ensures follow-up visits of 24 h and 7 days after EVT
- Radiologist: evaluates neuroimaging data
- Telephone assessor for long-term prognosis after EVT: conducts follow-up visits at 90 days after EVT by telephone interview
- Data manager: organizes data capture, processing and interpreting data, safeguards its quality and integrity
- Study coordinator: trial registration, coordinates study visits, refines the EDC system, collects adverse events, and reports to the safety supervisor
- Safety supervisor: screens for SAEs
- Steering committee: the Stroke Rapid Response Team members, resident research physicians, telephone assessors, study coordinators, and PI meet monthly to monitor the progress of the entire trial and, if in doubt, communicate promptly

No stakeholder or public group is involved.

#### Composition of the data monitoring committee, its role and reporting structure {21a}

There is no data monitoring committee in this study since this is not a masked study, and there is no requirement for protecting the blindness in this study. Another experienced independent neurosurgeon and anesthesiologist will be assigned to this study as a safety supervisor. In the case of SAEs, this safety supervisor will assess if the SAE is related to the intervention, and further treatment will be taken to ensure the participant’s safety.

#### Adverse event reporting and harms {22}

The research team continuously monitors the vital signs, adverse events, and SAEs for 24 h after EVT in the ICU and in the ward. Resident research physicians and evaluators will report the adverse events and SAEs to safety supervisors when managing patients or during each follow-up visit, and the PI will supervise and report adverse events to the steering committee and ethics committee within 24 h. The adverse events will be collected and reported by the standardized language of the Medical Dictionary for Regulatory Activities (MedDRA). All harms and adverse events will be reported in the publications.

#### Frequency and plans for auditing trial conduct {23}

Researchers will maintain study data by the requirements of the Good Clinical Practice. The study data will be submitted to the ethics committee every 3 months, and all information relevant to the study will be kept confidential for at least 5 years after the end of this study.

#### Plans for communicating important protocol amendments to relevant parties (e.g., trial participants, ethical committees) {25}

Potential amendments to the PPDET protocol are processed according to the Helsinki Declaration. The PI will be responsible for any decisions to modify the protocol. If the modification may affect the conduction of the study, or have potential impacts on the safety and interests of the participants, the PI will first report to the ethics committee and get approval before implementation and then update the trial information at ClinicalTrials.gov.

#### Dissemination plans {31a}

The results of our study will be disseminated in peer-reviewed journals. PI will review and approve each paper related to this study before submission. All the team members who have participated in this study for over 3 months will be included in the list of authors, otherwise will be listed in the acknowledgment.

## Discussion

Several previous studies have been carried out on intraoperative anesthetic management [16, 39–41]. However, there are no reports addressing the postoperative sedation after EVT and the duration of sedation.

This is the first randomized clinical trial to evaluate the efficacy and safety of postoperative prolonged sedation with DEX on the long-term prognosis of patients with AIS after successful reperfusion.

DEX and propofol are commonly used to relieve postoperative agitation for AIS patients after EVT. DEX has several merits in postoperative sedation when compared with propofol. First, DEX sedation could provide a more stable level of sedation than propofol [42, 43]. Although these previous studies were conducted with critically ill and mechanically ventilated patients and were limited by their small sample, they had unveiled the clinical possibility of postoperative prolonged sedation with DEX. Second, DEX has minimal respiratory depression specially referred to those patients under neurosurgery or with critical conditions [20]. Thus, we hypothesize that DEX may be the most applicable postoperative sedative for AIS patients after EVT. Third, higher SBP after successful reperfusion is related to an increased risk of intracerebral hemorrhage [13]. At the same time, the demerit of DEX-induced hypotension may benefit AIS patients to this point. Previous animal studies suggested the DEX post-treatment could provide neuroprotective effects in cerebral ischemia-reperfusion injury [27, 44, 45]. However, whether it would be effective in patients under EVT still remains unknown. So, we conduct this study to verify our hypothesis on whether postoperative prolonged sedation with DEX could provide consistent neuroprotection for AIS patients under EVT.

Continuously cerebral protection should not only focus on the intraoperative period but also include the postoperative period. Exploring a new time window to preserve neurological functions might provide potential clinical benefits to AIS patients. There are few studies related to postoperative sedation, let alone the duration. To explore the effect of postoperative sedation under a relatively sufficient duration, the DEX infusion is scheduled for 24 h following EVT.

There are several strengths of this study. This is the first study as far as we know to focus on the neuroprotective effects of DEX in the postoperative period. The effect of postoperative prolonged sedation is evaluated not only by functional outcomes (mRS at 90 days) but also with the neuroimaging assessment (changes of ischemic penumbra volume and infarct volume at 7 days when compared with baseline).

There are several limitations to this study. First, this is a single-site study, which reduces the generalizability of the findings. Second, this is an open-label trial without blinding the participants and the researchers conducting assessments in the hospital, as DEX could be easily distinguished by prolonged drowsiness after treatment. However, our team members responsible for mRS evaluations, statisticians, and radiologists are blinded to allocation.

In conclusion, postoperative prolonged sedation with DEX might promote the long-term prognosis of patients with AIS after successful reperfusion. It may propose a new pharmacological approach to improve the independence of stroke survivors and reduce the related socio-economic burden.

### Trial status

The study protocol was registered on June 7, 2021. The recruitment has yet to start. Recruitment is expected to end in December 2026.

### Supplementary Information

**Supplementary Material 1.**

**Supplementary Material 2.**

## Abbreviations

EVT: Endovascular thrombectomy
AIS: Acute ischemic stroke
DEX: Dexmedetomidine
mTICI: Modified thrombolysis in cerebral infarction
mRS: Modified Rankin Scale
NIHSS: National Institute of Health Stroke Scale
ICU: Intensive care unit
HR: Heart rate
BPV: Blood pressure variability
CT: Computed tomography
CTA: CT angiography
CTP: CT perfusion
BP: Blood pressure
SBP: Systolic blood pressure
DBP: Diastolic blood pressure
MAP: Mean arterial pressure
ECG: Electrocardiogram
MRI-DWI: Magnetic resonance imaging-diffusion weighted imaging
CTP-rCBF: CT angiography-relative cerebral blood flood
EDC: Electronic data capture
eCRF: Electronic case report form
ATMPs: Advanced therapy medicinal products
SAE: Serious adverse event
PI: Principal investigator
SD: Standard deviation
IQR: Interquartile range
RR: Relative risk
CI: Confidence interval
MedDRA: Medical Dictionary for Regulatory Activities
