# Supplementary material for: Effect of Postoperative Prolonged sedation with Dexmedetomidine after successful reperfusion with Endovascular Thrombectomy on long-term prognosis in patients with acute ischemic stroke (PPDET): study protocol for a randomized controlled trial
Source: Trials. 2024 Mar 4;25:166. doi: 10.1186/s13063-024-08015-x (PMC10913237; doi:10.1186/s13063-024-08015-x)
Supplement: Supplementary file 1 — Supplementary Material 1. [file 13063_2024_8015_MOESM1_ESM.pdf]

Appendix

Supplementary Table 1. The modified thrombolysis in cerebral infarction scale (mTICI)

Grade		Description
0		No perfusion
1		Antegrade reperfusion past the initial occlusion, but limited distal branch filling with little or slow distal reperfusion
2	2a	Antegrade reperfusion of less than half of the occluded target artery previously ischemic territory (e.g., in one major division of the middle cerebral artery [MCA] and its territory)
	2b	Antegrade reperfusion of more than half of the previously occluded target artery ischemic territory (e.g., in two major divisions of the MCA and their territories)
3		Complete antegrade reperfusion of the previously occluded target artery ischemic territory, with an absence of visualized occlusion in all distal branches

Supplementary Table 2. The modified Rankin Scale (mRS)

Grade	Description
0	No symptoms at all
1	No significant disability despite symptoms; able to carry out all usual duties and activities
2	Slight disability: unable to carry out all previous activities but able to look after own affairs without assistance
3	Moderate disability: requiring some help, but able to walk without assistance
4	Moderately severe disability: unable to walk without assistance, and unable to attend to own bodily needs without assistance
5	Severe disability: bedridden, incontinent, and requiring constant nursing care and attention
6	Death

Supplementary Table 3. National Institute of Health Stroke Scale (NIHSS)

Item	NIHSS	Score
1a	LOC	0 = Alert
		1 = Arousable by minor stimulation
		2 = Arousable by strong/repeated stimulation
		3 = Unresponsive
1b	LOC questions	0 = Answers both correctly
		1 = Answers one correctly
		2 = Answers neither correctly
1c	LOC commands	0 = Performs both tasks correctly
		1 = Performs one task correctly
		2 = Performs neither task
2	Gaze	0 = Normal
		1 = Partial gaze palsy
		2 = Forced deviation

3	Visual	0 = Normal<br>1 = Partial hemianopia<br>2 = Complete hemianopia<br>3 = Bilateral hemianopia
4	Facial Palsy	0 = Normal<br>1 = Minor<br>2 = Partial<br>3 = Complete
5a<br>5b<br>6a<br>6b	Motor Arm (left)<br>Motor Arm (right)<br>Motor Leg (left)<br>Motor Leg (right)	0 = No drift<br>1 = Drift before 10s<br>2 = Falls before 10s<br>3 = No effort against gravity<br>4 = No movement<br>UN = Amputation or joint fusion explain:
7	Limb Ataxia	0 = Absent<br>1 = In one limb<br>2 = In two limbs<br>UN = Amputation or joint fusion
8	Sensory	0 = Normal<br>1 = Mild-to-Moderate loss<br>2 = Severe-to-total loss
9	Language	0 = Normal<br>1 = Mild-to-Moderate aphasia<br>2 = Severe aphasia<br>3 = Mute or global aphasia
10	Dysarthria	0 = Normal<br>1 = Mild-to-Moderate<br>2 = Severe<br>UN = Intubated
11	Extinction and Inattention	0 = Normal<br>1 = Visual, tactile, auditory, spatial, or personal inattention<br>2 = Profound hemi-inattention or extinction to more than one modality

Supplementary Table 4. The Ramsay sedation score

Score	Description
1	Awake; agitated or restless or both
2	Awake; cooperative, oriented, and tranquil
3	Awake but responds to commands only
4	Asleep; brisk response to light glabellar tap or loud auditory stimulus
5	Asleep; sluggish response to light glabellar tap or loud auditory stimulus
6	Asleep; no response to glabellar tap or loud auditory stimulus
